# Saccharomyces cerevisiae derived postbiotic alters gut microbiome metabolism in the human distal colon resulting in immunomodulatory potential *in vitro*

**DOI:** 10.3389/fmicb.2024.1358456

**Published:** 2024-02-12

**Authors:** Cindy Duysburgh, Lisa Miclotte, Justin B. Green, Kevin T. Watts, Maria I. Sardi, Anirikh Chakrabarti, Ehsan Khafipour, Massimo Marzorati

**Affiliations:** ^1^ProDigest BV, Ghent, Belgium; ^2^Cargill, Ankeny, IA, United States; ^3^Cargill, Wayzata, MN, United States; ^4^Cargill, Plymouth, MN, United States; ^5^Cargill R&D Centre Europe, Vilvoorde, Belgium; ^6^Center of Microbial Ecology and Technology (CMET), Ghent University, Ghent, Belgium

**Keywords:** EpiCor, postbiotic, gut microbiota, SHIME, immunomodulation, gut health, *Bifidobacterium*

## Abstract

The yeast-based postbiotic EpiCor is a well-studied formulation, consisting of a complex mixture of bioactive molecules. In clinical studies, EpiCor postbiotic has been shown to reduce intestinal symptoms in a constipated population and support mucosal defense in healthy subjects. Anti-inflammatory potential and butyrogenic properties have been reported *in vitro*, suggesting a possible link between EpiCor’s gut modulatory activity and immunomodulation. The current study used a standardized *in vitro* gut model, the Simulator of the Human Intestinal Microbial Ecosystem (SHIME^®^), to obtain a deeper understanding on host-microbiome interactions and potential microbiome modulation following repeated EpiCor administration. It was observed that EpiCor induced a functional shift in carbohydrate fermentation patterns in the proximal colon environment. Epicor promoted an increased abundance of *Bifidobacterium* in both the proximal and distal colon, affecting overall microbial community structure. Co-occurrence network analysis at the phylum level provided additional evidence of changes in the functional properties of microbial community promoted by EpiCor, increasing positive associations between Actinobacteria with microbes belonging to the Firmicutes phylum. These results, together with a significant increase in butyrate production provide additional support of EpiCor benefits to gut health. Investigation of host-microbiome interactions confirmed the immunomodulatory potential of the applied test product. Specific microbial alterations were observed in the distal colon, with metabotyping indicating that specific metabolic pathways, such as bile acid and tryptophan metabolism, were affected following EpiCor supplementation. These results, especially considering many effects were seen distally, further strengthen the position of EpiCor as a postbiotic with health promoting functionality in the gut, which could be further assessed *in vivo*.

## 1 Introduction

The human gut microbiota has been indispensably linked with health and disease, with numerous studies exploring the effects of selective alteration of the intestinal microbiome in improving the health status of the host. Selective modulation of the gut microbiome can be achieved through strategies that include, but are not limited to, dietary supplementation with prebiotics, probiotics, or postbiotics (Liu et al., 2022). Postbiotics have been recently defined through a consensus of gut health scientists by the International Scientific Association for Probiotics and Prebiotics (ISAPP) as “a preparation of inanimate microorganisms and/or their components that confers a health benefit on the host” (Salminen et al., 2021). A postbiotic formulation may contain microbial cell components, microbial metabolites produced through anabolic activity of microbes, and intermediate or end-products of microbial fermentation produced through catabolic activity of microbes. This potentially gives postbiotics a wider variety of modes of action compared to other prebiotics and probiotics. Also, because these functions do not require viable microbes, postbiotics provide additional advantages over probiotics in supplement and functional food formulations that involve processes and ingredient matrices that are not hospitable to live microbe stability (Salminen et al., 2021). To date various postbiotics are produced from well-recognized microorganisms belonging to the *Lactobacillus* and *Bifidobacterium* genera. Daily administration of an inanimate form of *Bifidobacterium bifidum* MIMBb75, for instance, has been shown to alleviate gastrointestinal symptoms in irritable bowel syndrome (IBS) patients (Andresen et al., 2020), whereas an inanimate culture of *Lactobacillus gasseri* CP2305 was reported to positively regulate intestinal function by stimulating bifidobacterial abundances and reducing p-cresol levels amongst others in healthy human subjects (Sugawara et al., 2016). While the previous examples of postbiotics are produced by probiotic microorganisms, the ISAPP postbiotic definition does not require that the source microbial strain(s) of a postbiotic be known probiotic(s). *Akkermansia muciniphila*, for instance, improved several metabolic parameters in relation to diet-induced metabolic disorders when administered in an inanimate form, with efficacy even being enhanced as compared to administration of live *A. muciniphila* (Cani and de Vos, 2017; Depommier et al., 2019).

The potential mechanisms behind the ability of postbiotics to beneficially alter host health have been reviewed by Salminen et al. (2021), and are mainly associated with the presence of sufficient amounts of bioactive molecules. Important effector molecules that can be present in postbiotic formulations include organic acids, such as lactate and short-chain fatty acids (SCFA), which are able to beneficially affect human health by modulating gut microbial composition and/or enhancing gut barrier integrity. Indeed, microbially produced organic acids can exert antimicrobial effects against pathogens (Abdelhamid et al., 2018) and stimulate cross-feeding interactions resulting in the downstream production of health-beneficial metabolites (Moens et al., 2017), as well as increase transepithelial resistance (Feng et al., 2018). Immunomodulation has been recognized as another potential mechanism in which postbiotics could improve the health status of the host (Salminen et al., 2021; Liu et al., 2022). It has been reported that the microbially produced organic acid butyrate can suppress pro-inflammatory immune responses due to activation of immune signaling pathways (Thangaraju et al., 2009; Ji et al., 2016), though other microbial-derived compounds that could be present in postbiotic formulations could stimulate other immunomodulatory properties. Beta-glucans present in the yeast cell walls of *Saccharomyces cerevisiae*, for instance, have been reported to interact with several immune receptors in ovine cell culture assays (Jin et al., 2019), while a *S. cerevisiae* based postbiotic reduced the expression of interleukin (IL) 6, nuclear factor kappa-light-chain-enhancer of activated B cells (NF-kB) and IL-1b and increased levels of tight junction proteins in poultry (Chuang et al., 2021), confirming the potential of postbiotics to be involved in immunomodulation and enhancement of gut barrier integrity. Similarly in ruminants, *S. cerevisiae* based postbiotics have been shown to prime the immune system and reduce inflammation during pathogenic (Mahmoud et al., 2020; Vailati-Riboni et al., 2021), physiological (Li et al., 2016; Guo et al., 2022), and immunological challenges (Jiang et al., 2018).

The yeast postbiotic EpiCor is a clinically tested proprietary formulation that has been well-studied over the past decade (Jensen et al., 2007, 2008; Moyad et al., 2009; Evans et al., 2012; Possemiers et al., 2013; Pinheiro et al., 2017; Ducray et al., 2019). EpiCor postbiotic is a nutrient-rich product fermented from the baker’s yeast variant of *S. cerevisiae* using proprietary methods and fermentation media. It contains a complex profile of bioactive molecules, including vitamins, polyphenols, phospholipids and yeast cell wall components such as beta-glucans, mannans and other polysaccharides (Jensen et al., 2008; Ducray et al., 2016). A possible link between the action of the yeast postbiotic in the gut and immunomodulatory properties is under investigation, as EpiCor has been shown to exert significant anti-inflammatory activity and selectively enhance butyrate production *in vitro* (Jensen et al., 2007; Possemiers et al., 2013) as well as support mucosal defense in human clinical trials (Jensen et al., 2008). Furthermore, Pinheiro et al. (2017) have reported that daily dosing of 500 mg EpiCor modulated gut microbial composition in human subjects experiencing gastrointestinal discomfort and constipation, resulting in the improvement of intestinal symptoms, such as reduced levels of bloating and distension.

The aim of the current study was to further explore the impact of repeated daily doses of EpiCor in the human intestine in relation to gut health and immunity. For this purpose, the Simulator of the Human Intestinal Microbial Ecosystem (SHIME^®^), a standardized *in vitro* simulator of the human gastrointestinal tract (Van de Wiele et al., 2015; Van den Abbeele et al., 2018a), was used to assess the effects of EpiCor on microbial activity and composition along the entire colon. To obtain a deeper view on the potential alterations of the gut microbiome following EpiCor administration, long-read shotgun metagenomic sequencing and liquid chromatography mass spectrometry (LC-MS)-based metabolomics were employed, while also assessing effects on gut barrier function and immune regulation using co-cultures of Caco-2 cells and THP-1 macrophages (Daguet et al., 2016).

## 2 Materials and methods

### 2.1 Ethics

The study was conducted in accordance with the Declaration of Helsinki, and approved by the Ethics Committee of the University Hospital Ghent (reference number B670201836585).

### 2.2 Experimental design of the SHIME^®^ model

The effects of EpiCor postbiotic on the gut fermentation characteristics, and gut microbiome and metabolome were investigated making use of the SHIME^®^ model (Van de Wiele et al., 2015), a dynamic semi-continuous reactor-based model that mimics the different stages of the human gastrointestinal tract (ProDigest-Ghent University, Ghent, Belgium). EpiCor was provided by Cargill (USA) and tested at an *in vitro* dose of 500 mg per day, an equivalent dose in an adult human.

The reactor configuration for this experiment was adapted from the setup described by Molly et al. (1993), and consisted of a succession of three reactors simulating the different regions of the gastrointestinal tract, i.e., upper gastrointestinal tract including subsequent simulation of stomach and small intestine, proximal colon (PC; pH = 5.75–5.95; 500 mL) and distal colon (DC; pH = 6.6–6.9; 800 mL), respectively. At the start of the experiment, the colonic reactor vessels were inoculated with the fecal material from one healthy human donor, who had not received any antibiotic treatment in the 4 months prior to donation. Inoculum preparation, feeding regime, temperature settings and reactor feed composition were previously described by Possemiers et al. (2004) and adopted during the current study. To maintain anoxic conditions, all reactors of the SHIME^®^ setup were flushed once every day for 5 min with N_2_-gas.

The timeline of the SHIME^®^ -experiment comprised a 2-week stabilization period, followed by a 2-week control period and a 3-week treatment period (Supplementary Figure 1). During the stabilization phase, the gut microbiota was allowed to differentiate in the different reactors depending on the local environmental conditions, while during the control period the baseline microbial activity, and during the 3-week treatment period, the effect of repeated daily EpiCor administration on the microbiome was investigated. To assess fermentation characteristics, microbiome composition and function, and metabolomic profile, samples were collected from each colonic reactor vessel (PC and DC) three times per week during the 2-week control period (*n* = 3 samples/week × 2 weeks = 6/reactor) and 3-week treatment period (*n* = 3 samples/week × 3 weeks = 9/reactor).

### 2.3 Fermentation characteristics

Determination of SCFA concentrations, including acetate, propionate, butyrate and branched chain fatty acids (bCFA; sum of isobutyrate, isovalerate, and isocaproate) was executed as previously described by De Weirdt et al. (2010). Lactate levels were determined using a commercially available enzymatic assay kit (R-Biopharm, Darmstadt, Germany) according to manufacturer’s instructions and measured on an iCubio iMagic-M9 (Shenzhen iCubio Biomedical Technology Co. Ltd., Shenzhen, China). Ammonium analysis was run on an AQ300 Discrete Analyzer (Seal-Analytical, Rijen, Netherlands) using the indophenol blue spectrophotometric method according to manufacturer’s instructions.

### 2.4 Microbiome composition and function

DNA was isolated as previously described by Van den Abbeele et al. (2018b), starting from pelleted cells originating from 1 mL SHIME^®^ samples, with minor modifications. A bead-beating step was included using the NGS PréMax Homogenizer device (BioSPX, Abcoude, Netherlands), thereby performing the homogenization procedure twice for 40 s at 6 m/s with the sample being allowed to rest for 5 min between shakings. Following extraction, DNA concentrations were determined using the Quant-iT kit (Thermo Fisher Scientific, USA) following manufacturer’s instructions. Next, libraries were constructed with the SQK-RPB004 Rapid PCR Barcoding kit (Oxford Nanopore Technology (ONT), Oxford, UK). Blanks were included during library preparation for quality control. Shotgun metagenomic sequencing was performed using R9.4.1 FLO-MIN 106 flow cells on the GridION platform (ONT, Oxford, UK), multiplexing 12 samples in each flow cell. Each sequencing run lasted 70 h. MinKNOW ONT software (v 4.2.5) with Guppy basecaller (v 4.3.4) was used for sequencing using the high-accuracy basecalling setting, followed by de-multiplexing, adapter trimming, and quality control using default settings. The sequence data can be found at: https://www.ncbi.nlm.nih.gov/, Submission ID: SUB13991422 under BioProject ID: PRJNA1045629.

Fastq files obtained from the MinKNOW ONT workflow were used for microbial taxonomic classification. First, host DNA was removed by mapping fastq files to the human genome using Minimap2 (Li, 2018) followed by the removal of any reads matching the host genome using Samtools (Li et al., 2009). Remaining reads were assumed to be from microbial DNA and used for taxonomic assignation, which was performed using Kraken2 (Wood et al., 2019) with a custom database containing high quality genomes from RefSeq database (Tatusova et al., 2014), and published metagenome-assembled genomes (Almeida and De Martinis, 2021; Hiseni et al., 2021), classified according to the Genome Database Taxonomy (Chaumeil et al., 2019). Estimation of abundance at different taxonomic levels (i.e., species, genus, family, and phylum) was done with Bracken (Bayesian Reestimation of Abundance with KrakEN) (Lu et al., 2017). First, a Bracken file was created from our customized Kraken database using a read length flag of 500. Bracken was then run to estimate abundance at multiple taxonomic levels from the Kraken report, using default settings.

To assess microbiome functional potential, we identified gene orthologs annotated by KEGG (Kanehisa et al., 2004), and carbohydrate-active enzymes (CAZy) (Drula et al., 2022). First, genomes from microbial species identified with Kraken2 with relative abundance higher than 10^–4^ were annotated using PROKKA (Seemann, 2014), followed by additional assessment of gene function using EggNOG-mapper v2 (Cantalapiedra et al., 2021). After the annotation process was completed, KEGG Orthology genes (KOs) and CAZys were compiled for each genome, weighted based on the presence of each species abundance within the community, generating the accumulated KEGG KO and CAZy potential for all the microbes identified in each sample.

### 2.5 Metabolomics

First, laser-assisted rapid evaporative ionization mass spectrometry (LA-REIMS) metabolic fingerprinting was performed as a first-line rapid discriminative metabotyping to inform decision making for which samples to select for additional in-depth LC-MS-based metabolomics. Briefly, 200 μL sample was transferred into a well of a 96-well plate and subjected to LA-REIMS analysis as described by Van Meulebroek et al. (2020).

Next, in-depth LC-MS-based metabolomics, including ultra-high pressure liquid chromatography high-resolution mass spectrometry (UHPLC-HRMS) analysis of samples for polar metabolomics and lipidomics was performed on a subset of the collected samples, including three samples collected during the final control week and five samples collected during the final treatment weeks from the DC reactor. For polar metabolomics analysis, samples were subjected to a liquid extraction protocol and subsequent instrumental analysis based on the work of Vanden Bussche et al. (2015) and De Paepe et al. (2018). For lipidomics analysis, samples were subjected to a liquid-liquid extraction protocol and subsequent instrumental analysis as described by Van Meulebroek et al. (2017). Prior to analysis, the MS system was calibrated according to manufacturer’s guidelines (Thermo Fisher Scientific, USA) to warrant accurate mass measurements (<5 ppm mass deviation) in both positive and negative ionization mode. In addition, the chromatographic and MS performances were evaluated by injecting standard mixtures that contained the target metabolites. Furthermore, an internal quality control (iQC) sample was assembled by combining equal shares from the extracts that were obtained from the collected samples and employed to condition the LC-MS instrument as well as to perform continuous monitoring of the instrument performance by repeated analysis of the iQC-sample throughout the sequence. A metabolite was positively identified in the LC-MS raw data taking into account the (i) accurate m/z-value of the molecular ion (allowed mass deviation of 5 ppm), (ii) 13C isotope pattern, and (iii) relative retention time (taking into account the retention time of the nearest eluting internal standard with an allowed time deviation of 2.5%). For identification and quantification of metabolites, Xcalibur software (v 4.0.27.21; Thermo Fisher Scientific, USA) was used. In general, a metabolite was considered below the limit of detection/quantification if the metabolite’s observed peak area was below 250,000 arbitrary units (AU). Following peak integration, the area ratio was determined for each metabolite by calculating the ratio between the area of the metabolite and that of the most suited internal standard. Besides the sample-specific normalization that was based on the internal standard, an intra-batch normalization strategy was also applied based on the iQC samples.

### 2.6 Caco-2/THP1-blue™ co-culture model

To measure the potential gut barrier strengthening and immunomodulatory effects of EpiCor, an intestinal co-culture model consisting of Caco-2 and THP1-cells was exposed to samples collected at the end of the control period and at the end of the treatment period from each colonic reactor vessel of the SHIME^®^ experiment. Transepithelial electrical resistance (TEER) was used as a measure for gut barrier integrity, while measurements of NF-kB and cytokines [IL-6, IL-10, IL-1b, IL-8, tumor necroses factor (TNF)-alpha, Cys-X-Cys motif chemokine ligand (CXCL) 10, and monocyte chemoattractant protein (MCP)-1] activity were performed to determine immunomodulatory effects. The co-culture was set up and the different measurements performed as previously described (Ghyselinck et al., 2021). As reported by Ghyselinck et al. (2021) experimental control were included in the setup, using a medium control when investigating TEER and a control in which cells were stimulated at the basolateral side with Caco-2 complete medium containing ultrapure LPS (LPS + control) for the other parameters.

### 2.7 Data processing and statistics

Comparison of data obtained on fermentation characteristics for control and treatment periods was performed by means of Wilcoxon Rank Sum tests in R. Differences were considered significant if *p* < 0.05 and were considered tendencies if 0.05 < *p* < 0.1.

Before analyzing the microbiome composition data, rare genera with relative abundance less than 10^–5^ were removed, decreasing the dataset from 193 to 133 genera. Alpha diversity metrics (i.e., Shannon indices) were calculated in R using the Phyloseq package (McMurdie and Holmes, 2013) using species count tables from Bracken as input normalized using rarefaction. Statistical analysis for alpha diversity metrics was performed with the lme4 package from R, using the fit linear mixed-effect model function lmer (De Boeck et al., 2011) with the formula: ∼ Treatment × Colon region. This model was used to test for differences in the microbiome between control and treatment samples for the PC and DC. Beta diversity was performed with Principal Component Analysis (PCA) of centered log-ratio (CLR) transformed genus counts using the Phyloseq package. Beta diversity was tested with Permutational Multivariate Analysis of Variance (PERMANOVA) using the Adonis function in the vegan package. The model included Aitchison distances (Aitchison et al., 2000) calculated based on CLR transformed values as the dependent variable (Anderson, 2001), assessing associations between treatment, colon region, and their interaction with the composition of the microbiome. Differential abundance analysis was performed with the R package LinDA (Zhou et al., 2022) which was modified to save each individual model for every taxon, CAZy gene family, and KEGG KO, rather than only the coefficient estimates to allow for the use of more complex *post hoc* contrasts using the R package emmeans (Lenth, 2022). The same models used in the alpha diversity analyses were used in the differential abundance analysis. The emmeans package was used to conduct one *post hoc* pairwise comparisons after the models were fit, comparing treatments in a pairwise method at each colon region separately. *P*-values were corrected for multiple hypothesis testing using the Benjamini-Hochberg method (Benjamini and Hochberg, 2018). Overall, differences were considered significant if *p* < 0.01. To assess ecological dynamics, microbial network analyses were performed with the R package NetCoMi (Peschel et al., 2021) using phylum level microbiome data. First, rare species with relative abundance less than 10^–4^ were removed from the analysis. Network construction was normalized using CLR and limited the taxa that was present in a minimum of 75% of samples, using an absolute Pearson correlation ≥0.5. This was done separately for PC and DC samples. Each comparison was done using the “netCompare” function.

The LA-REIMS data were first processed using MassLynx^®^ V4.1 Progenesis^®^ Bridge (Waters Corporation, UK) whereby extracted ion chromatograms were created as well as background subtraction was performed. Following ion chromatogram extraction, bridged data were processed by Progenesis^®^ QI V2.3 (Waters Corporation, UK) in order to list the detected m/z features and their relative abundances. To this end, the automatic sensitivity mode for peak picking (default value) was applied. The abundances of the detected m/z features were normalized based on the total ion abundance. Normalized data were subjected to multivariate statistical analysis using SIMCA^®^ version 17 (Sartorius, Germany). Here, data were pre-processed, including log-transformation to induce normal distributions and unit variance scaling (1/standard deviation) to standardize the range of signal intensities. Unsupervised principal component analysis (PCA-X) was executed to assess the natural patterning of samples and reveal potential outliers (based on the Hoteling’s T^2^ criterion). Orthogonal partial least squares discriminant analysis (OPLS-DA) was used to differentiate samples according to experimental conditions in a supervised fashion. Validity of the OPLS-DA models was verified by permutation testing (*n* = 100), cross-validated analysis of variance (*p* < 0.05), and the quality parameter Q^2^(Y) (≥0.5).

Processing of the metabolite data obtained using the in-depth LC-MS-based metabolomics approach was founded on a sequential approach of multivariate and univariate statistical analysis. As part of the applied multivariate statistical analysis, PCA-X and OPLS-DA modeling were performed using SIMCA^®^ version 17. Validity of the OPLS-DA models was assessed based on the same three parameters as used above. If all three parameters were compliant with the set criteria, the model was considered valid and key metabolites that were responsible for the separation between the investigated groups were selected based on the variable importance in projection score (VIP-value), Jack-knifed confidence interval, and S-plot coordinates. Selected metabolites were then subjected to univariate statistical analysis. As a preliminary check for univariate statistics, normality of relative abundances was evaluated using a Shapiro-Wilk test (*p* < 0.05) and a Quantile-Quantile plot for every metabolite of interest. Since many of the detected metabolites showed deviations from normality, non-parametric tests were used during further data analysis, i.e., Wilcoxon rank-sum tests (*p* < 0.05).

Finally, to evaluate differences in TEER and immune markers between the control and treatment, a two-way ANOVA with Sidak’s multiple comparisons test was performed using GraphPad Prism version 9.1.2 for Windows (GraphPad Software, San Diego, CA, USA), with differences considered significant if *p* < 0.05.

## 3 Results

### 3.1 Effects on fermentation characteristics

**FIGURE 1:**
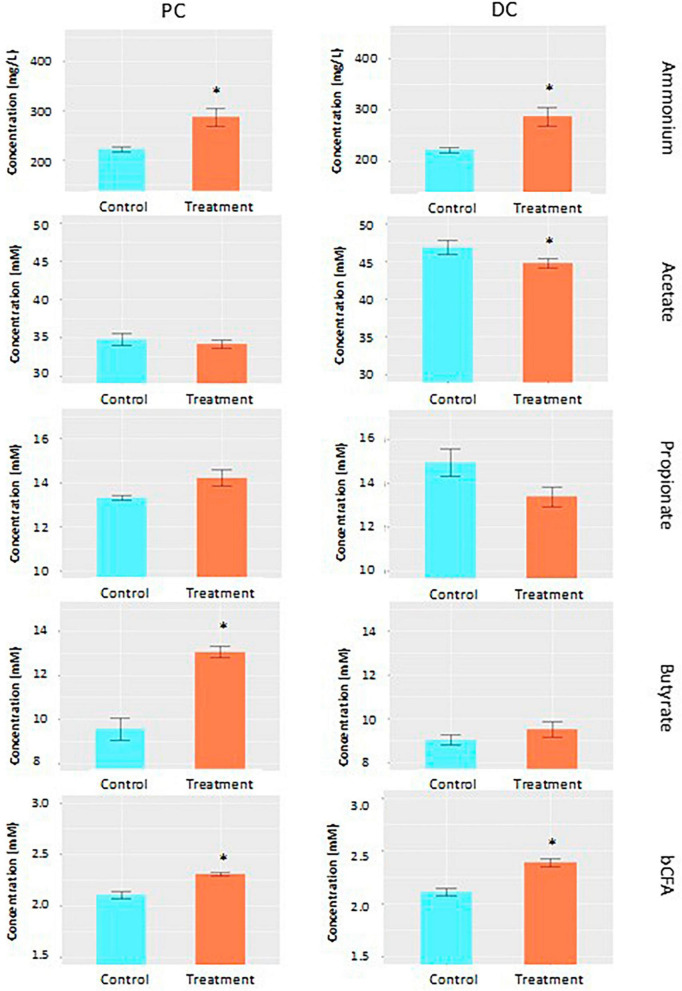
Fermentation characteristics. Average acetate (mM), propionate (mM), butyrate (mM), ammonium (mg/L) and branched chain fatty acid (bCFA; mM) levels during the control (*n* = 3 samples/week × 2 weeks = 6) and treatment (*n* = 3 samples/week × 3 weeks = 9) periods in the proximal (PC) and distal colon (DC) reactors of the SHIME^®^ -experiment following daily treatment with EpiCor postbiotic. Data is presented as mean ± SEM. Statistically significant differences relative to the control period are indicated with *p < 0.05.

Average across weeks within control and treatment periods, acetate levels slightly decreased during treatment compared to the control in both colonic regions (−1.9 and −4.5% in PC and DC, respectively), though only reaching significance in DC (*p* = 0.012) (Figure 1 and Supplementary Table 1). Butyrate levels, on the other hand, were increased in both colonic regions upon EpiCor supplementation compared to the control period (+ 36.7% and + 5.3% in PC and DC, respectively), with strongest effects observed in PC, reaching significance in that colonic region (*p* = 0.0004). Administration of Epicor tended to change the propionate production in both colonic regions (*p* = 0.088 and *p* = 0.050 in PC and DC, respectively), with increased propionate levels in PC (+ 6.7%), but reduced levels in DC (−10.4%). The bCFA and ammonium concentrations were significantly increased following repeated administration with EpiCor compared to the control period (*p* = 0.0004 for bCFA in PC and DC; *p* = 0.0008 and *p* = 0.0004 for ammonium in PC and DC, respectively).

### 3.2 Effects on microbial community composition

**FIGURE 2:**
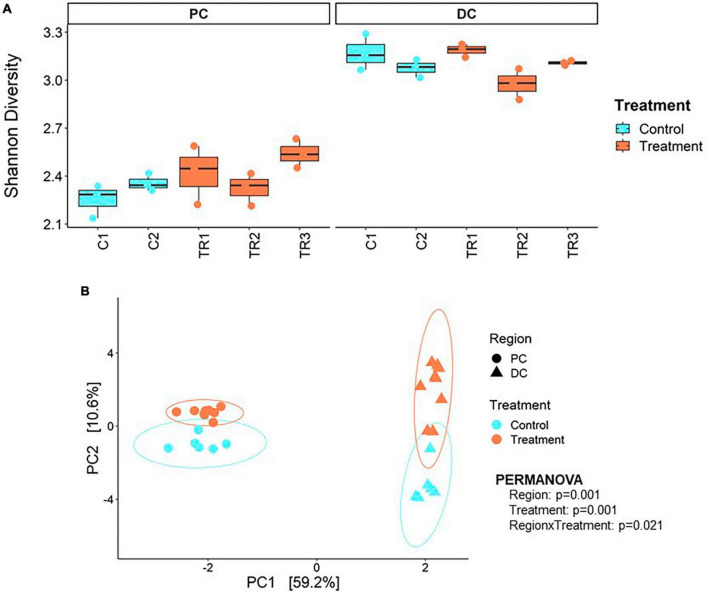
Microbial alpha and beta diversity. **(A)** Shannon alpha diversity at microbial species level and **(B)** principal component analysis (PCA) at microbial genus level performed on the samples collected during the 2 weeks control (C1–C2; *n* = 3 samples/week × 2 weeks = 6) and 3 weeks treatment (TR1-3; *n* = 3 samples/week × 3 weeks = 9) in the proximal (PC) and distal colon (DC) reactors of the SHIME^®^ -experiment following treatment with EpiCor postbiotic.

Average across weeks within the control period, Shannon alpha diversity indices of 2.31 ± 0.09 and 3.12 ± 0.10 were observed in PC and DC regions, respectively (Figure 2A). Following treatment with EpiCor, alpha diversity tended to increase in PC (2.43 ± 0.15; *p* = 0.057) but was not affected in DC (3.09 ± 0.11; *p* = 0.0607).

**FIGURE 3:**
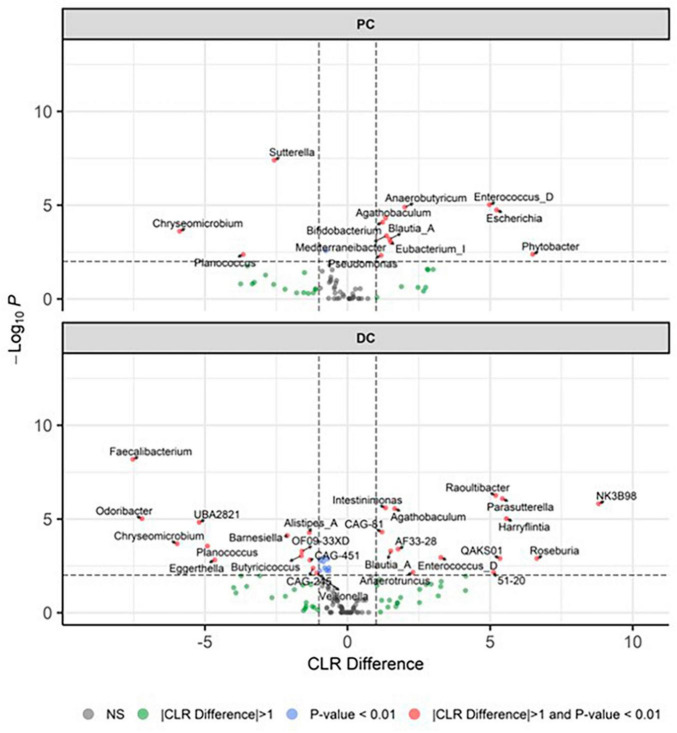
Differentially abundant genera. Presented as volcano plots, showing the significantly (*p* < 0.01) altered genera during the control (*n* = 3 samples/week × 2 weeks = 6) and treatment (*n* = 3 samples/week × 3 weeks = 9) periods in the proximal (PC) and distal colon (DC) reactors of the SHIME^®^ -experiment, with a positive CLR difference indicating a higher abundance following treatment with EpiCor postbiotic compared to the control. Horizontal dotted lines represent the defined cut-off for statistical significance (*p* = 0.01), while vertical dotted lines represent the defined cut-off for biologically relevant changes (—CLR difference— = 1).

Beta diversity revealed distinct clustering patterns of samples based on PC and DC regions, and between control period and EpiCor treatment (Figure 2B). The first principal components explained 59.2% of the variability, separating samples by region, while the second principal component, which explained 10.6% of the variability, separated samples by treatment. The PERMANOVA analysis confirmed above observations (Region, *p* = 0.001; Treatment, *p* = 0.001, Region × Treatment, *p* = 0.021), demonstrating EpiCor differentially affected microbiome composition in PC vs. DC. Differential abundance analysis (Figure 3) identified significantly (*p* < 0.01) stimulated bacterial genera following EpiCor supplementation. Significant genera that were affected by a CLR difference > 1 included *Bifidobacterium*, *Phytobacter*, *Escherichia*, *Anaerobutyricum*, *Mediterraneibacter*, *Eubacterium_I*, and *Pseudomonas* in the PC; *Intestinimonas*, *Harryflintia*, *Parasutterella*, *Roseburia*, *Anaerotruncus*, *Raoultibacter*, *NK3B98*, *QAKS01*, *51–20*, *AF33-28*, and *CAG-81* in the DC; and *Blautia_A*, *Enterococcus_D*, and *Agathobaculum* in both the PC and DC. On the other hand, the *Planococcus* and *Chryseomicrobium* genera were consistently and significantly more abundant during the control compared to the treatment period in both colonic regions, while *Sutterella* was statistically reduced following repeated EpiCor administration in the PC, with similar effects observed for *Alistipes_A*, *Barnesiella*, *Butyricicoccus*, *Eggerthella*, *Faecalibacterium*, *Odoribacter*, *Veillonella*, *CAG-451*, *CAG-245*, *UBA2821*, and *OF09-33XD* in the DC. It was observed that most treatment effects were already established during the first week following EpiCor exposure (Supplementary Figures 2–5). However, while for *Roseburia* and *Anaerotruncus* an initial stimulation was observed in the DC following EpiCor supplementation, their abundance reduced during the final weeks of treatment (Supplementary Figures 2–5), indicating gradual establishment of novel cross-feeding mechanisms following repeated administration of the EpiCor postbiotic. Furthermore, when considering the time effects during the SHIME^®^ experiment (Supplementary Figures 2–5), the observed treatment effects on *Pseudomonas* and *Blautia_A* in the PC, and *Harryflintia* and *CAG-245* in the DC were less evident, as a linear increase over time was observed throughout the complete experimental run.

**FIGURE 4:**
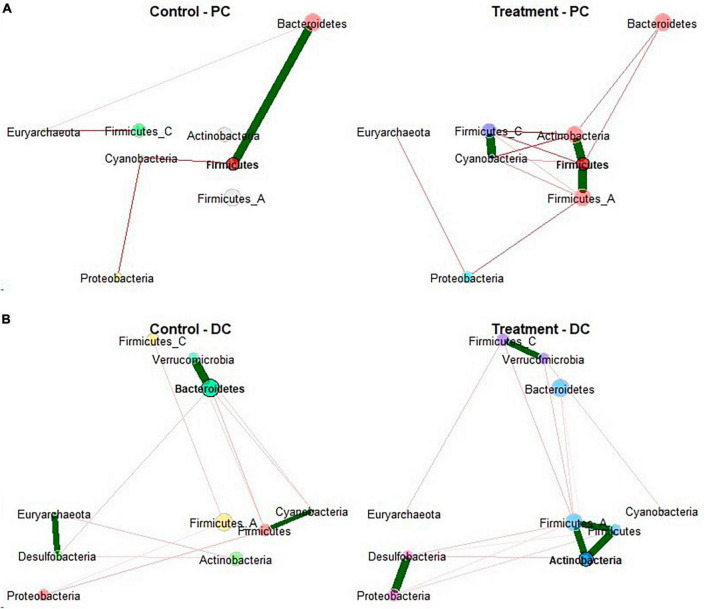
Microbial network analysis. Network construction and comparison of metagenomic microbiome data performed on the samples collected during the control (*n* = 3 samples/week × 2 weeks = 6) and treatment (*n* = 3 samples/week × 3 weeks = 9) period in the **(A)** proximal (PC) and **(B)** distal colon (DC) reactors of the SHIME^®^ -experiment following treatment with EpiCor postbiotic. Node colors represent bacterial phyla with their size as an indicator of their centrality. Each edge represents a significant positive (green line) or negative (red line) interaction between nodes. The thickness of edge represents the strength of correlation.

To further explore the effect EpiCor on the structure of microbial communities, microbiome correlation networks were constructed to determine whether treatment with EpiCor was affecting cross-feeding interactions between bacterial groups in the community (Figure 4). This network analysis confirmed that the PC microbiome was significantly more interconnected following treatment with EpiCor as compared to the control microbiome in this colonic area (*p* = 0.021). A specific trend concerned the enhanced microbial interaction of the Actinobacteria phylum with the microbial community in both colonic regions, especially showing strong interactions with the Firmicutes phylum.

**FIGURE 5:**
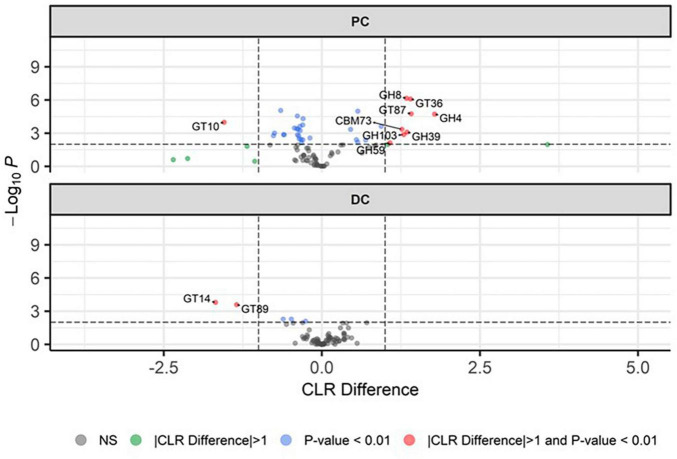
Microbial functional potential with respect to carbohydrate active enzymes. Differential abundance analysis, presented as volcano plots, showing the significantly (*p* < 0.01) altered carbohydrate active enzymes (CAZy) during the control (*n* = 3 samples/week × 2 weeks = 6) and treatment (*n* = 3 samples/week × 3 weeks = 9) period in the proximal (PC) and distal colon (DC) reactors of the SHIME^®^ -experiment, with a positive CLR difference indicating a higher abundance following treatment with EpiCor postbiotic compared to the control. Horizontal dotted lines represent the defined cut-off for statistical significance (*p* = 0.01), while vertical dotted lines represent the defined cut-off for biologically relevant changes (|CLR difference| = 1).

Upon identifying the CAZy potential of the microbiome (Figure 5), it was observed that the profile of enzymes that cleave complex carbohydrates shifted following EpiCor supplementation, with the strongest significant effects observed in PC, while only limited statistical differences were found between the control and treatment period in DC. Stimulated CAZy in PC following EpiCor treatment included glycoside hydrolase families (GH) 4, GH8, GH39, GH59, GH103, glycosyl transferase families (GT) 36 [re-classified in the CAZy database (Drula et al., 2022) to GH94], GT87 and carbohydrate-binding module families (CBM) 73, with strongest effects (indicated by the highest CLR difference) observed for GH4, GT36, and GT87. On the other hand, GT10 was significantly reduced in PC with the same observations for GT14 and GT89 in DC following repeated EpiCor administration.

At the gene level, we found 395 KEGG KOs with significant differences in PC (*p* < 0.01), while no significant differences were found between the control and treatment period in DC. Gene functions with increase abundance in the PC during the treatment period included 24 ABC transporters, and genes involved in starch and sucrose metabolism, propionate, butyrate, and purine metabolism. Conversely, during the control period in the PC, we observed increase abundance of genes involved in biosynthesis of amino acids, and glycine, serine, and threonine metabolism (Supplementary Table 2).

### 3.3 Effects on metabolomic fingerprinting

**FIGURE 6:**
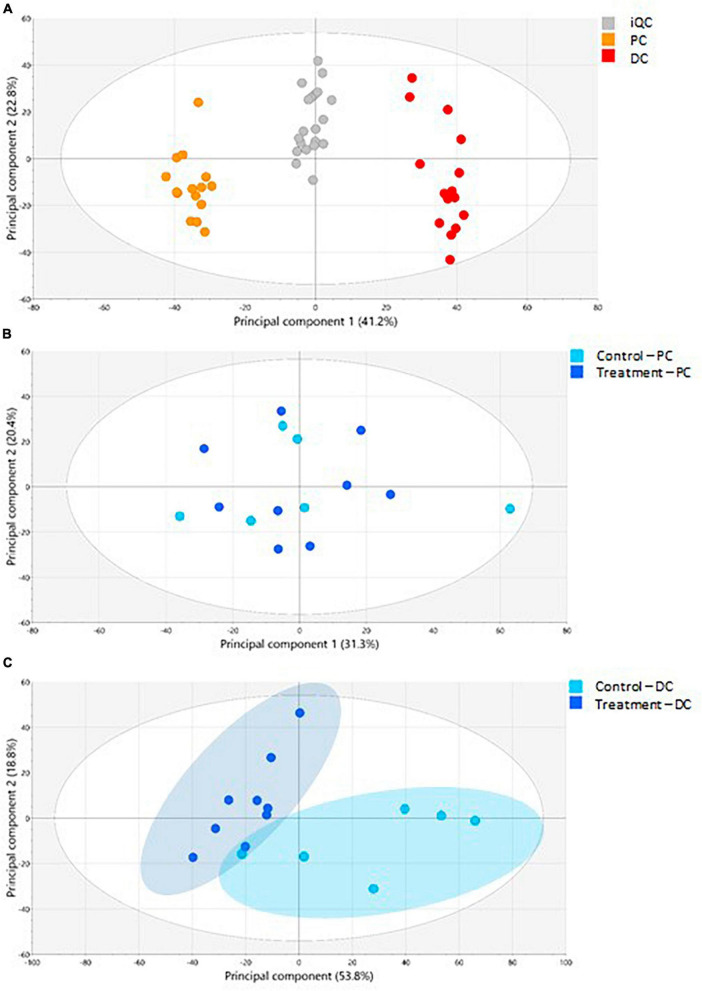
The LA-REIMS profiling in negative ionization mode. **(A)** The PCA-X score plot for the iQC-samples as well as the samples collected during the 2 weeks control (*n* = 3 samples/week × 2 weeks = 6) and 3 weeks treatment with EpiCor (*n* = 3 samples/week × 3 weeks = 9) in the proximal (PC) and distal colon (DC) reactors of the SHIME^®^ -experiment. Three sample clusters were observed, related to the PC samples (orange), DC samples (red), and iQC-samples (gray). **(B)** The PCA-X score plot for the PC samples (*n* = 15). Coloring of samples was conducted according to the control (light blue) and treatment (dark blue) period. **(C)** The PCA-X score plot for the DC samples (*n* = 15). Coloring of samples was conducted according to the control (light blue) and treatment (dark blue) period, with two sample clusters being observed.

The PCA-X modeling was performed to assess the natural patterning of the SHIME^®^ samples and define the analytical performance of the LA-REIMS analysis (Figure 6). The PCA-X model was constructed based on the SHIME^®^ samples as well as the iQC samples and was composed out of 12 principal components, whereby the first two principal components explained together 64.0% of the present variance (41.2% was enclosed by the first principal component) (Figure 6A). Three sample clusters were noted, of which one was related to the iQC samples and two to the SHIME^®^ samples, which related to the simulated PC and DC regions. When investigating the colon regions separately, it was observed that PCA-X modeling for the PC samples (6 principal components, with the first two explaining 51.7% of the variance) did not show a clear metabolic impact of EpiCor treatment (Figure 6B). Additional investigation of the treatment effect was achieved through supervised OPLS-DA modeling whereby no valid model could be generated for the comparison of EpiCor treatment with its control period. The PCA-X modeling for the DC samples (6 principal components, with the first two explaining 72.6% of the variance) revealed a clear differentiation between the control and treatment periods (Figure 6C). This was confirmed with OPLS-DA modeling, where a significant metabolic impact of EpiCor treatment was observed (*R*^2^X 0.776, *R*^2^Y 0.950, Q^2^Y 0.764, *p* = 0.0308).

The metabolic impact of EpiCor in the DC was further assessed using UHPLC-HRMS polar and lipidomics profiling (Supplementary Figure 6). A PCA-X model was constructed, whereby metabolic shifts were observed in both the polar (Supplementary Figure 6A) and lipidomics (Supplementary Figure 6B) profiling following treatment with EpiCor compared to the respective control period. The OPLS-DA modeling confirmed these findings (*R*^2^X 0.704, *R*^2^Y 1, Q^2^Y 0.974, *p* = 0.0104 for the polar metabolites; *R*^2^X 0.490, *R*^2^Y 0.888, Q^2^Y 0.821, *p* = 0.0135 for the lipids). Upon assessing the metabolites that were responsible for the observed significant metabolic segregations, univariate Wilcoxon-Mann-Whitney tests showed that the levels of 46 metabolites significantly altered following EpiCor supplementation compared to the control period (Table 1). Of these metabolites, 9 were decreased and 37 were increased following repeated EpiCor administration. Several metabolites from the tryptophan metabolism pathway were significantly altered, with 3-hydroxyanthranilic acid, 3-hydroxykynurenine, kynurenine, nicotinamide, tryptamine, and xanthurenic acid being stimulated following EpiCor supplementation, while the combined measurement of nicotinic acid and picolinic acid was reduced. Metabolic shifts were also observed with respect to carbohydrate homeostasis, with EpiCor treatment resulting in increased levels of 2-aminoadipic acid, lactose, maltose, sucrose and trehalose, and reduced levels of xylose. Furthermore, bile acid metabolism was affected following EpiCor supplementation, as shown by significant reductions of cholic acid (CA) and 7-ketodeoxycholic acid, while ursodeoxycholic acid (UDCA) levels were enhanced. Finally, other metabolites that were significantly affected and showed at least a twofold change following treatment with EpiCor, included 1,3-propanediol, 3,4-dihydroxyphenylacetic acid, 4-hydroxyproline, 4-methyl-2-oxovaleric acid, acetylcarnitine, dehydroisoandrosterone, glutaric acid, hippuric acid, malonic acid, malonylcarnitine, alanine, and sphinganine, with the latter two compounds being elevated in the control period, while the former metabolites were enhanced during the treatment period.

**TABLE 1:** Discriminating metabolites in the distal colon.

Metabolite	Relative abundance (AU)		*p*-value
	**Control**	**Treatment**	
2-Piperidinone	3.045	3.926	0.0357
Cholic acid	5.594	2.360	0.0357
7-Ketodeoxycholic acid	6.061	2.259	0.0357
UDCA	0.875	1.263	0.0357
Choline	0.916	1.181	0.0357
Sphinganine	1.556	0.734	0.0357
2-Hydroxyhexanoic acid	0.274	0.485	0.0357
Pantothenic acid	0.156	0.299	0.0357
Undecanedioic acid	0.204	0.297	0.0357
Nicotinic acid–Picolinic acid	0.839	0.276	0.0357
4-Hydroxy-proline	0.092	0.242	0.0357
Malic acid	0.306	0.234	0.0357
N-acetyl-glutamic acid	0.109	0.200	0.0357
N-acetyl-proline	0.081	0.132	0.0357
Kynurenine	0.094	0.124	0.0357
Nicotinamide	0.045	0.108	0.0357
Hippuric acid	0.036	0.100	0.0357
N-acetyl-alanine	0.146	0.082	0.0357
Tryptamine	0.066	0.073	0.0357
1,3-Propanediol	0.020	0.045	0.0357
Malonic acid	0.016	0.040	0.0357
4-Methyl-2-oxovaleric acid	0.013	0.035	0.0357
3-Hydroxyanthranilic acid	0.003	0.027	0.0357
Putrescine	0.014	0.023	0.0357
Aspartic acid	0.011	0.017	0.0357
Xanthurenic acid	0.005	0.015	0.0358
4-Methylcatechol	0.010	0.015	0.0357
Uridine	0.024	0.015	0.0357
3,4-Dihydroxy phenylacetic acid	0.003	0.011	0.0325
2-Aminoadipic acid	0.004	0.010	0.0357
Thymine	0.008	0.010	0.0357
Acetylcarnitine	0.000	0.010	0.0358
Dehydroisoan drosterone	0.001	0.009	0.0358
Xylose	0.057	0.009	0.0357
Adenosine-5-monophosphate	0.006	0.008	0.0357
Alanine	0.028	0.007	0.0357
Lactose	0.001	0.007	0.0357
Maltose	0.001	0.007	0.0357
Sucrose	0.001	0.007	0.0357
Threonic acid	0.003	0.006	0.0357
3-Methyl-2-oxobutyric acid	0.003	0.005	0.0357
N-acetyl-methionine	0.004	0.005	0.0357
Trehalose	0.002	0.004	0.0357
3-Hydroxykynurenine	0.001	0.002	0.0358
Malonylcarnitine	0.000	0.002	0.0357
Glutaric acid	0.001	0.001	0.0357

Average relative abundance [arbitrary units (AU)] obtained collected during the control (*n* = 3 samples during second week = 3) and treatment (*n* = 2 samples during second week and 3 samples during third week = 5) periods in the distal colon (DC) reactors of the SHIME^®^ -experiment following treatment with EpiCor postbiotic. Univariate statistics were based on Wilcoxon-Mann-Whitney testing with *p*-values indicated in the table.

### 3.4 Effects on immunomodulation and barrier integrity

**FIGURE 7:**
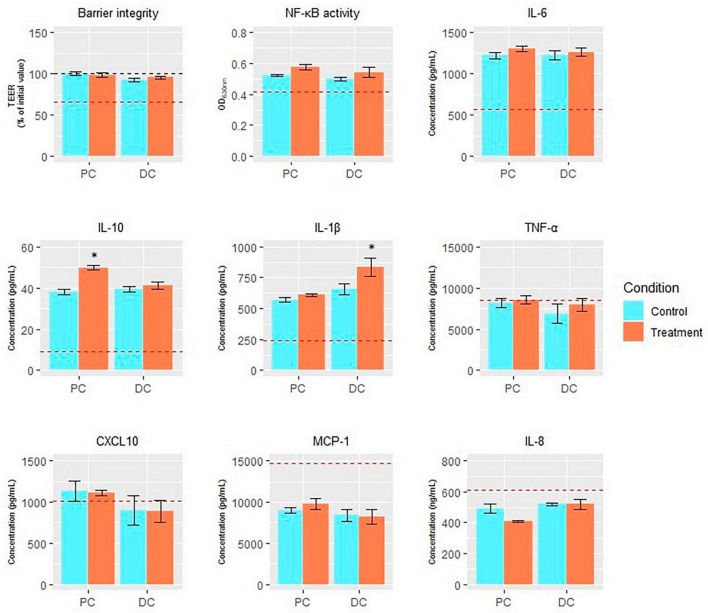
Endpoints of Caco-2/THP1-Blue™ co-culture model. Average transepithelial electrical resistance (TEER;% of initial value), NF-κB activity (OD_630__nm_), and levels of IL-6 (pg/mL), IL-10 (pg/mL), IL-1β (pg/mL), TNF-α (pg/mL), CXCL10 (pg/mL), MCP-1 (pg/mL), and IL-8 (ng/mL) in the Caco-2/THP-1-Blue™ co-cultures conducted on samples taken at the end of the control (*n* = 1) and treatment (*n* = 1) periods in the proximal and distal colon reactors of the SHIME^®^ -experiment following treatment with EpiCor postbiotic. The red dotted line corresponds to the experimental control (medium control for TEER and LPS + control for the other parameters). Data are plotted as mean ± SEM (*n* = 3; technical replicates). Statistically significant differences relative to the control period are indicated with *p < 0.05.

When assessing effects of EpiCor on intestinal barrier integrity *in vitro* (Figure 7), it was observed that all colonic SHIME^®^ samples increased TEER compared to the experimental control, keeping its levels at the initial values, with no additional protective effects being observed following repeated EpiCor administration.

Furthermore, most immune markers (except TNF-alpha, MCP-1 and IL-8) were stimulated following administration of the colonic SHIME^®^ samples (from both the control and treatment period) to the Caco-2/THP1-Blue™ co-culture compared to the experimental control (Figure 7). Treatment with EpiCor only numerically increased NF-kB activity compared to the control period in each colon region. Furthermore, secretion of IL-10 (*p* = 0.043) and IL-1b (*p* = 0.004) was significantly stimulated in the PC and DC, respectively, following repeated EpiCor administration.

## 4 Discussion

During the performed SHIME^®^ study, the effects of repeated daily administration of the yeast postbiotic EpiCor was assessed on gut fermentation characteristics, microbiome and metabolome, and barrier function and immune modulation *in vitro*. The initial inoculum was obtained from a healthy volunteer and EpiCor postbiotic was supplemented at a dose of 500 mg per day, which is equivalent to the test dose applied in clinical trials (Pinheiro et al., 2017).

Supplementation of the EpiCor postbiotic increased the alpha diversity of microbiome in the PC region. This increase in diversity was accompanied with a shift in the microbiome composition (beta diversity) in the PC region. In particular, EpiCor postbiotic induced a significant increase in *Bifidobacterium* abundance in the PC, with species from this bacterial genus being known to be highly involved in saccharolytic degradation processes (Pokusaeva et al., 2011). Functional genomic profiling indeed showed a shift in the carbohydrate-fermenting potential of the microbiome in the PC following treatment with the yeast postbiotic, mainly stimulating several GH enzymes, which are involved in the hydrolysis of glycosidic bonds, at the expense of GT10 (a galactoside α-1,3/1,4-L-fucosyltransferase), with strongest effects observed for GH4 and GH94. While the GH94 family encompasses several phosphorylase enzymes, GH4 is in a family including several enzymes displaying an unusual mechanism of action involving NAD + . Both GH4 and GH94 activity have been associated with several bacterial groups within the human microbiome, including (but not limited to) *Bifidobacterium*, *Blautia*, *Escherichia*, *Enterococcus*, *Eubacterium*, and *Pseudomonas* for both GH94 and GH103, and *Phytobacter* for GH4, genera which were all significantly stimulated in the PC following treatment with EpiCor in the current study (Drula et al., 2022). Altogether, these data confirm that repeated EpiCor administration stimulated the microbial community involved in carbohydrate breakdown in the proximal colonic environment. The specific bifidogenic effect of EpiCor was confirmed by previously reported studies (Possemiers et al., 2013). Furthermore, *in vitro* experiments have shown that supplementation of beta-glucans derived from yeast cell walls enhanced the specific growth of *Bifidobacterium* species, especially *Bifidobacterium longum* (Wang H. et al., 2020) and *Bifidobacterium breve* (Keung et al., 2017). Furthermore, mannan-oligosaccharides, another important component in the outer cell wall of *S. cerevisiae*, have been shown to increase *Bifidobacterium* levels, though mainly in *in vivo* animal studies (Strickling et al., 2000; Grieshop et al., 2004; Teng and Kim, 2018). As many *Bifidobacterium* species have been considered probiotics (Chen et al., 2021), the selective stimulation of this bacterial group following EpiCor supplementation indicates a potential to positively impact gut health.

The proximal enrichment of the *Bifidobacterium* genus further stimulated cross-feeding interactions with other microbial groups, as shown by the significant butyrogenic effect in PC. This observation was confirmed by microbial network analysis, showing enhanced microbial interactions between the Actinobacteria (encompassing the *Bifidobacterium* genus) and Firmicutes phylum, the latter containing several butyrate-producing bacterial groups, following repeated EpiCor administration (Singh et al., 2023). Cross-feeding mechanisms between bifidobacteria and potent butyrate-producing microorganisms have been reported by multiple researchers (Rios-Covian et al., 2015; Moens et al., 2016; Rivière et al., 2016). Moens et al. (2016), for instance, reported specific cross-feeding interactions between different *Bifidobacterium* species and the butyrate-producing *Faecalibacterium prausnitzii* when providing the prebiotic inulin as a single nutrient source, while Bhattacharya et al. (2022) observed metabolic interactions between *Roseburia hominis* and *Bifidobacterium adolescentis* upon dosing beta-mannan-oligosaccharides. Furthermore, the observed butyrogenic potential of EpiCor confirmed findings obtained in previous studies. Possemiers et al. (2013) showed that repeated administration of EpiCor at a concentration of 630 mg/d enhanced butyrate production by the human gut microbiome *in vitro*, especially in the DC areas, with the results of the current study indicating that application of a lower dose (500 mg/d) can be as effective. In rats, supplementation of the yeast fermentate product stimulated butyrate-producing microorganisms, resulting in the prevention of adverse effects (such as reduced intestinal barrier integrity) induced by heat stress (Ducray et al., 2019). As butyrate is recognized as one of the main energy sources for the gut epithelium, and has shown high anti-inflammatory potential (van der Beek et al., 2017), the observed butyrogenic effect following EpiCor supplementation therefore further strengthens its position as a postbiotic beneficially affecting gut health. It must be mentioned that markers of proteolytic fermentation (bCFA and ammonium), which have been linked with adverse health effects, significantly increased following treatment with EpiCor. However, when comparing the obtained concentrations with values obtained in the intestinal lumen of the human colon of healthy individuals, bCFA and ammonium levels remained within the physiological ranges (Fried et al., 2017; Levitt and Levitt, 2019; Rios-Covian et al., 2020) and would therefore probably not contribute to any adverse health effect upon EpiCor supplementation.

Upon further assessment of microbiome changes following repeated EpiCor administration, specific microbial shifts could be observed in DC. Indeed, the significant interaction between colonic region and treatment implied that the level of shift in microbiome composition as a result of EpiCor supplementation was greater in the DC region compared to the PC. Strongly affected bacterial genera that were significantly stimulated in DC following treatment with EpiCor, included *Blautia*, *Intestimonas*, *Parasutterella*, *Anaerotruncus*, *Raoultibacter*, *Agathobaculum*, *Enterococcus*, and *Roseburia*. Several of these bacterial genera have been reported to be stimulated following supplementation of yeast-based postbiotics. The abundance of *Blautia*, for instance, was increased following administration of yeast mannoproteins during an *in vitro* trial (Li et al., 2022), while a high yeast dietary supplement resulted in significantly enhanced *Blautia* and *Roseburia* levels in piglets (Kiros et al., 2019). *Blautia* species are mainly involved in the fermentation of carbohydrates resulting in the production of acetate, lactate and ethanol (Liu et al., 2008) and have been linked with improved glucose metabolism in animal models (Yang et al., 2016). *Roseburia*, on the other hand, is a potent butyrate-producing bacterial genus, which has been positively correlated with anti-inflammatory properties (Ruan et al., 2022). Interestingly, the observed increase in *Roseburia* abundance following EpiCor supplementation during the current study was consistent with previous *in vitro* (Possemiers et al., 2013) and *in vivo* (Ducray et al., 2019) studies, though counteracting the findings of Pinheiro et al. (2017). However, in the latter study, the effect of the yeast fermentate on constipated individuals was evaluated, a population known to have a dysbiosed microbial community (Zhu et al., 2014). Indeed, similar as in Pinheiro et al. (2017), supplementation of EpiCor improved community composition. While in the previous study stimulation of *Bacteroides* and *Prevotella* species, which are bacterial groups that are typically depleted in constipated patients (Vandeputte et al., 2016), was observed, the current study showed increased *Roseburia* abundance at the expense of another butyrate-producing bacterial genus, i.e., *Faecalibacterium*, indicating a specific intensified cross-feeding between *Bifidobacterium* and *Roseburia* following EpiCor treatment.

Further in-depth assessment of the gut metabolome confirmed specific shifts in the DC region following supplementation of the yeast postbiotic. For instance, EpiCor proved to affect bile acid metabolism, which could be linked with changes in microbial community composition. Indeed, a significant reduction of 7-ketodeoxycholic acid was observed during the treatment period in the DC, which could be associated with decreased *Bacteroides* abundance, as this bacterial group is known to be highly involved in the biosynthesis of 7-ketodeoxycholic acid (Heinken et al., 2019). Furthermore, reduced CA levels were observed following EpiCor supplementation, while the levels of UDCA was enhanced. In the colon, primary bile acids like CA can be transformed into secondary bile acids through 7α- and/or 7β-dehydroxylation resulting in the production of deoxycholic acid, lithocholic acid and UDCA. This microbial activity can only be exerted by a few species in the gut microbiome, including some *Clostridium* species (Staley et al., 2017; Winston and Theriot, 2020). In the current study, the abundance of the *Clostridium* genus was, however, not significantly enhanced in the DC following repeated EpiCor administration, indicating that the potential stimulation of specific *Clostridium* species linking with the enhancement of the secondary bile acid UDCA was probably counteracted by the reduction of other species within the same genus. The production of secondary bile acids has been linked with several health promoting effects, as it can for instance serve as a colonization resistance against *Clostridioides difficile* infection (Winston and Theriot, 2016). In addition, several metabolites involved in intestinal tryptophan metabolism were significantly enhanced following repeated EpiCor administration, with the affected metabolites mainly belonging to the kynurenine pathway. This metabolic pathway has been linked with immunomodulation, with metabolites like xanthurenic acid, which was increased in the current study following EpiCor treatment, being shown to have anti-inflammatory and neuroprotective properties (Michaudel et al., 2022). Furthermore, EpiCor was also able to stimulate metabolites like acetylcarnitine and dehydroisoandrosterone, which have been linked with antioxidant and anti-inflammatory properties in animals and/or humans (Cao et al., 2020; Wang S. et al., 2020). These potential immunomodulatory properties of EpiCor (as observed through stimulation of specific metabolites) were further confirmed using *in vitro* cell culture assays, with EpiCor treatment resulting in a significant stimulation of IL-10 and IL-1b secretion in the PC and DC, respectively. While IL-1b is a pro-inflammatory cytokine (Ren and Torres, 2009), IL-10 has been linked with anti-inflammatory properties (Couper et al., 2008). Furthermore, previous studies evaluating the effect of EpiCor confirm its anti-inflammatory potential. Possemiers et al. (2013) for instance showed reduced pro-inflammatory IL-8 secretion during similar *in vitro* studies. Overall, these observed findings further strengthen the evidence for EpiCor’s postbiotic effect, showing its ability to not only affect gut health, but also immune health.

As with any *in vitro* study, the main limitation of the current work is that the observed effects cannot be directly translated to an *in vivo* biological response. Though, the obtained data support further clinical study of EpiCor for the enhancement of human health, including intestinal and immunomodulatory benefits through its effects on the gut microbiome. In conclusion, repeated administration of EpiCor postbiotic stimulated specific cross-feeding interactions between *Bifidobacterium* and the butyrate-producing *Roseburia* genus, resulting in a consistent butyrogenic effect *in vitro*. Next to the demonstration of immunomodulatory potential of EpiCor, a significant impact on the gut metabolome and community composition could specifically be observed in DC. This further strengthens the position of the yeast postbiotic due to its health-promoting effects in DC as many gastrointestinal diseases occur in this colonic region. Future studies are now warranted to confirm the observed postbiotic properties in humans, focusing on gut and immune health, while considering inter-individual variability.

## Data availability statement

The datasets presented in this study can be found in online repositories. The names of the repository/repositories and accession number(s) can be found below: NCBI–PRJNA1045629.

## Ethics statement

The studies involving humans were approved by the Ethics Committee of the University Hospital Ghent (reference number B670201836585). The studies were conducted in accordance with the local legislation and institutional requirements. The participants provided their written informed consent to participate in this study.

## Author contributions

MM: Conceptualization, Writing – review and editing. CD: Conceptualization, Data curation, Formal Analysis, Methodology, Writing – original draft. LM: Data curation, Formal Analysis, Writing – original draft. JG: Conceptualization, Funding acquisition, Writing – review and editing. KW: Conceptualization, Funding acquisition, Writing – review and editing. MS: Formal Analysis, Methodology, Writing – review and editing. AC: Data curation, Formal Analysis, Writing – review and editing. EK: Conceptualization, Data curation, Formal Analysis, Writing – review and editing.
