# Supplementary material for: Saccharomyces cerevisiae derived postbiotic alters gut microbiome metabolism in the human distal colon resulting in immunomodulatory potential in vitro
Source: Front Microbiol. 2024 Feb 12;15:1358456. doi: 10.3389/fmicb.2024.1358456 (PMC10895063; doi:10.3389/fmicb.2024.1358456)
Supplement: Supplementary file 2 [file Data_Sheet_1.PDF]

Supplementary Material

1 Supplementary Figures and Tables

1.1 Supplementary Figures

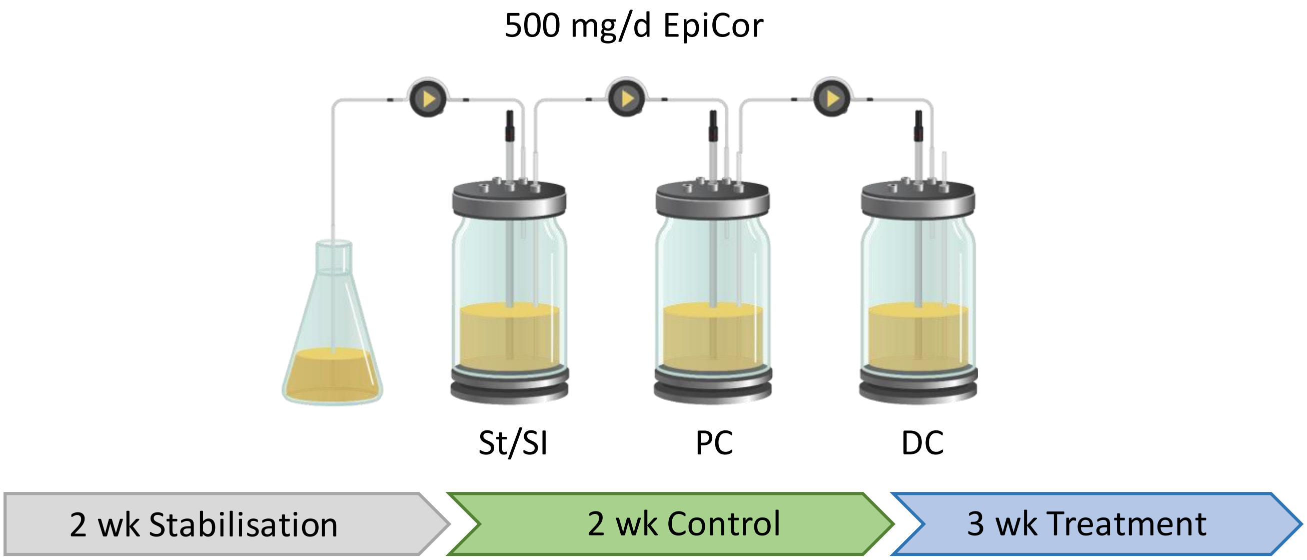

Supplementary Figure 1. Overview of experimental setup and timeline of the SHIME®-experiment. EpiCor™ postbiotic (500 mg/d) was applied as a treatment during the treatment phase. St/SI: stomach/small intestine; PC proximal colon; DC: distal colon.

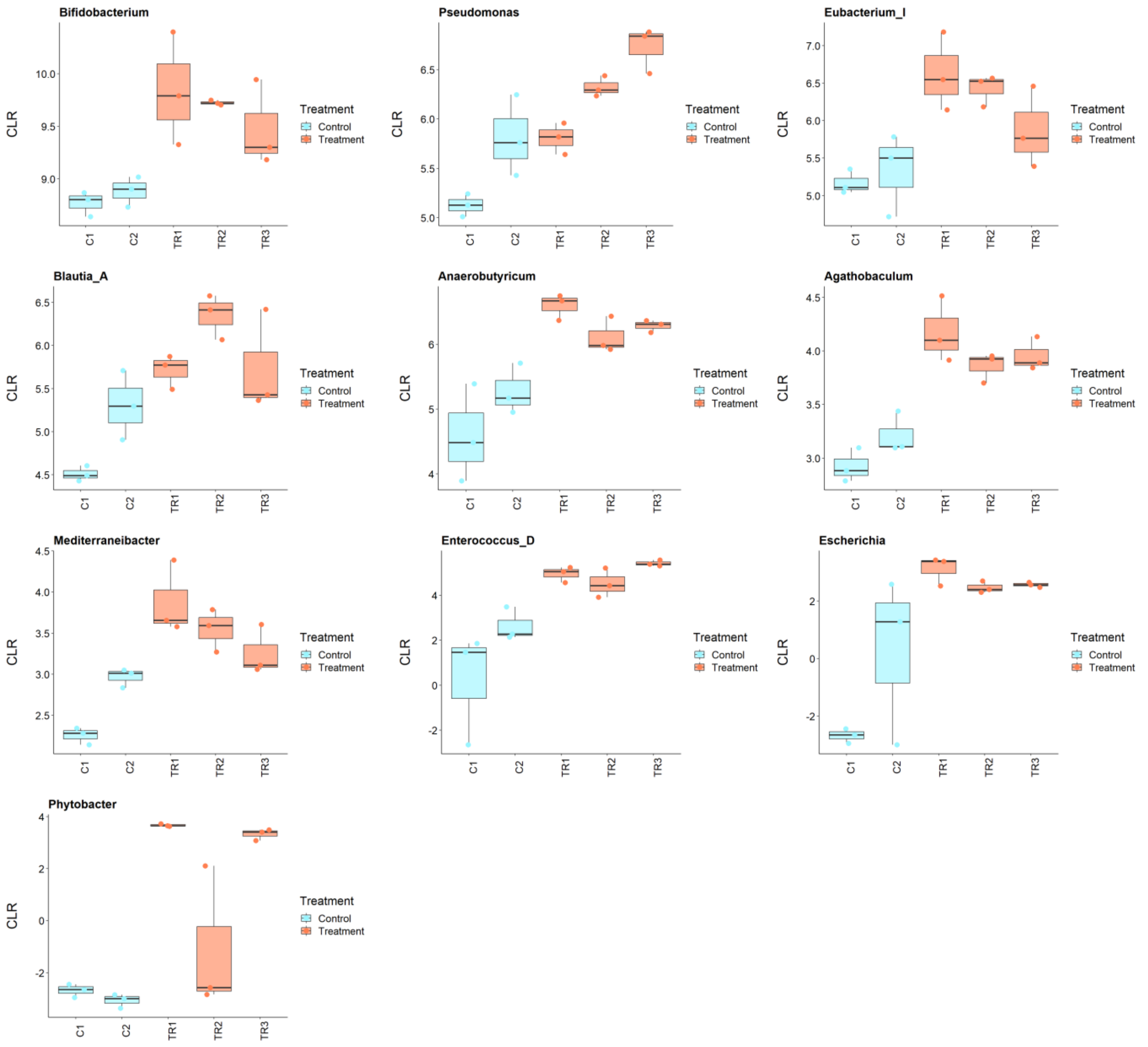

Supplementary Figure 2. Enhanced genera in proximal colon following EpiCor postbiotic treatment. Box plots generated for the significantly enhanced bacterial genera following treatment with EpiCor postbiotic as seen by differential abundance analysis, showing centered log-ratio (CLR) transformed data during the two weeks control (C1-2; n = 3 samples/week x 2 weeks = 6) and three weeks treatment (TR1-3; n = 3 samples/week x 3 weeks = 9) in the proximal colon (PC) reactors of the SHIME®-experiment.

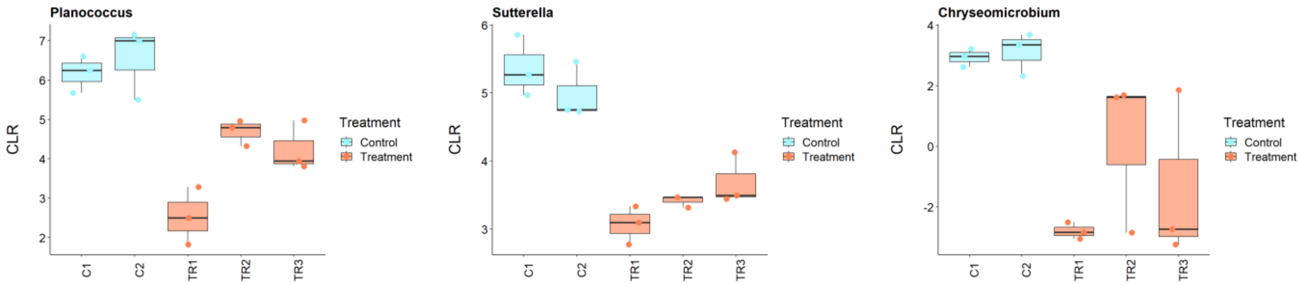

Supplementary Figure 3. Reduced genera in proximal colon following EpiCor postbiotic treatment. Box plots generated for the significantly reduced bacterial genera following treatment with EpiCor postbiotic as seen by differential abundance analysis, showing centered log-ratio (CLR) transformed data during the two weeks control (C1-2; n = 3 samples/week x 2 weeks = 6) and three weeks treatment (TR1-3; n = 3 samples/week x 3 weeks = 9) in the proximal colon (PC) reactors of the SHIME®-experiment.

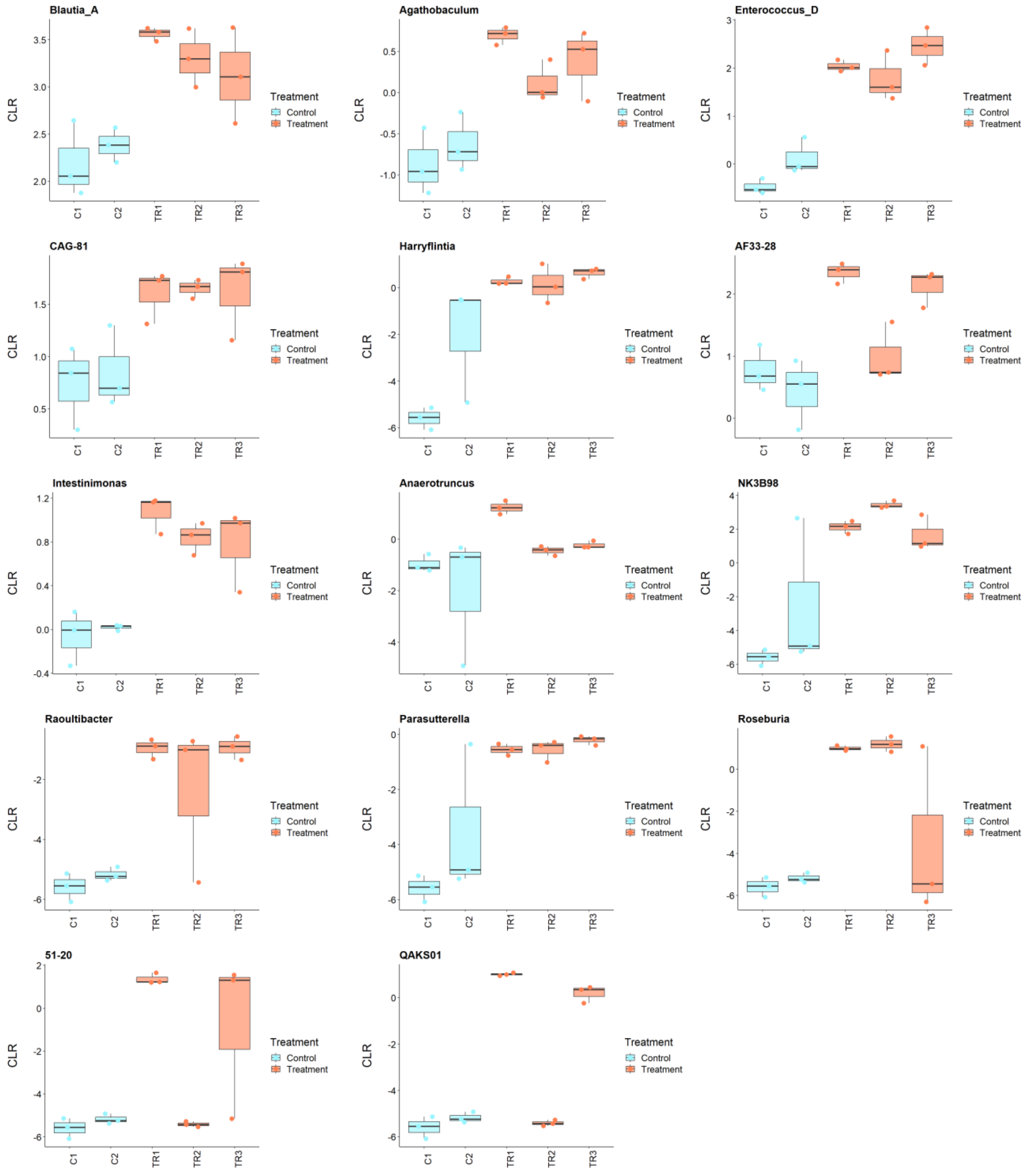

Supplementary Figure 4. Enhanced genera in distal colon following EpiCor postbiotic treatment. Box plots generated for the significantly enhanced bacterial genera following treatment with EpiCor postbiotic as seen by differential abundance analysis, showing centered log-ratio (CLR) transformed data during the two weeks control (C1-2; n = 3 samples/week x 2 weeks = 6) and three weeks treatment (TR1-3; n = 3 samples/week x 3 weeks = 9) in the distal colon (DC) reactors of the SHIME®-experiment.

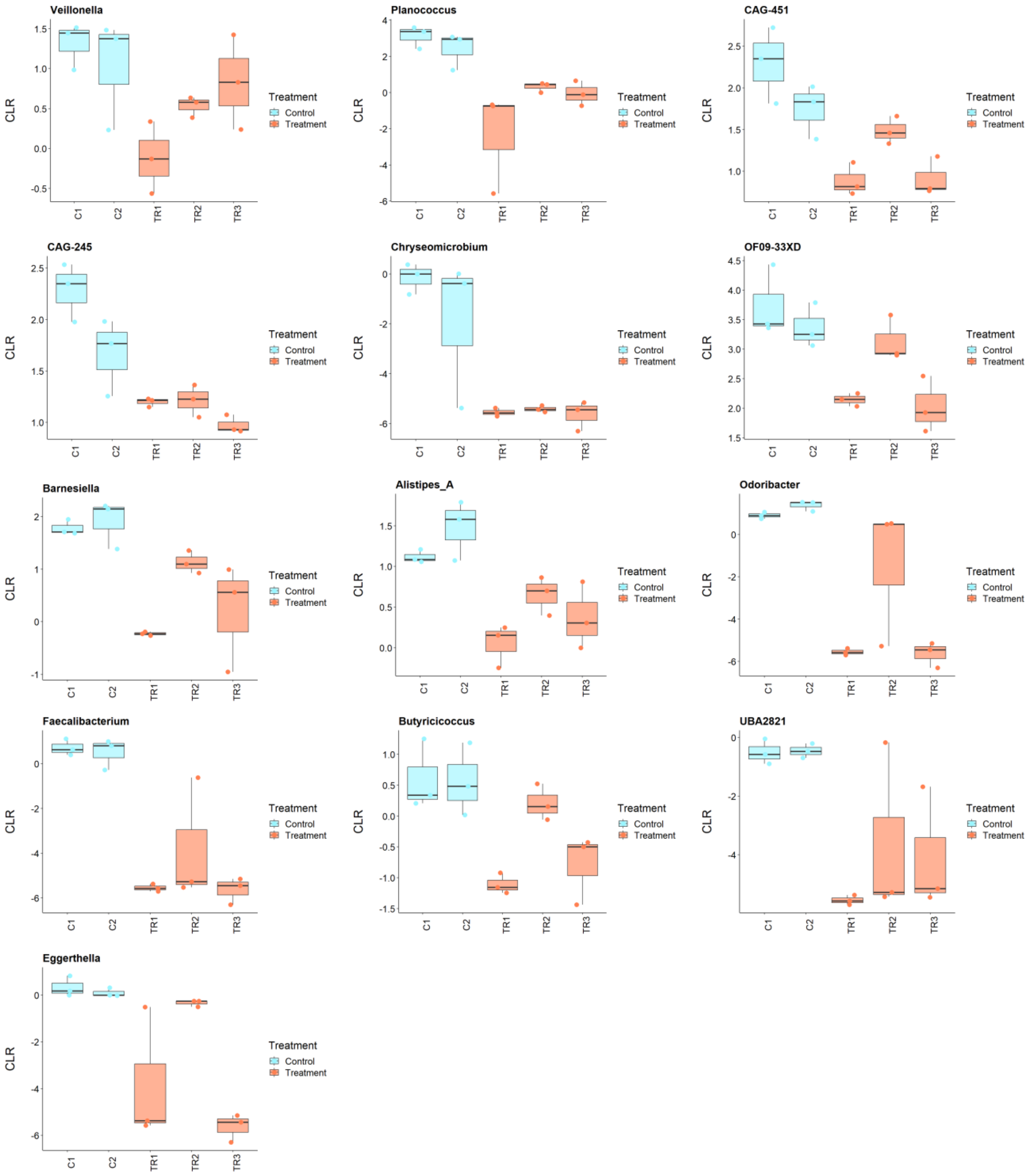

Supplementary Figure 5. Reduced genera in distal colon following EpiCor postbiotic treatment. Box plots generated for the significantly reduced bacterial genera following treatment with EpiCor postbiotic as seen by differential abundance analysis, showing centered log-ratio (CLR) transformed data during the two weeks control (C1-2; n = 3 samples/week x 2 weeks = 6) and three weeks treatment (TR1-3; n = 3 samples/week x 3 weeks = 9) in the distal colon (DC) reactors of the SHIME®-experiment.

A

B

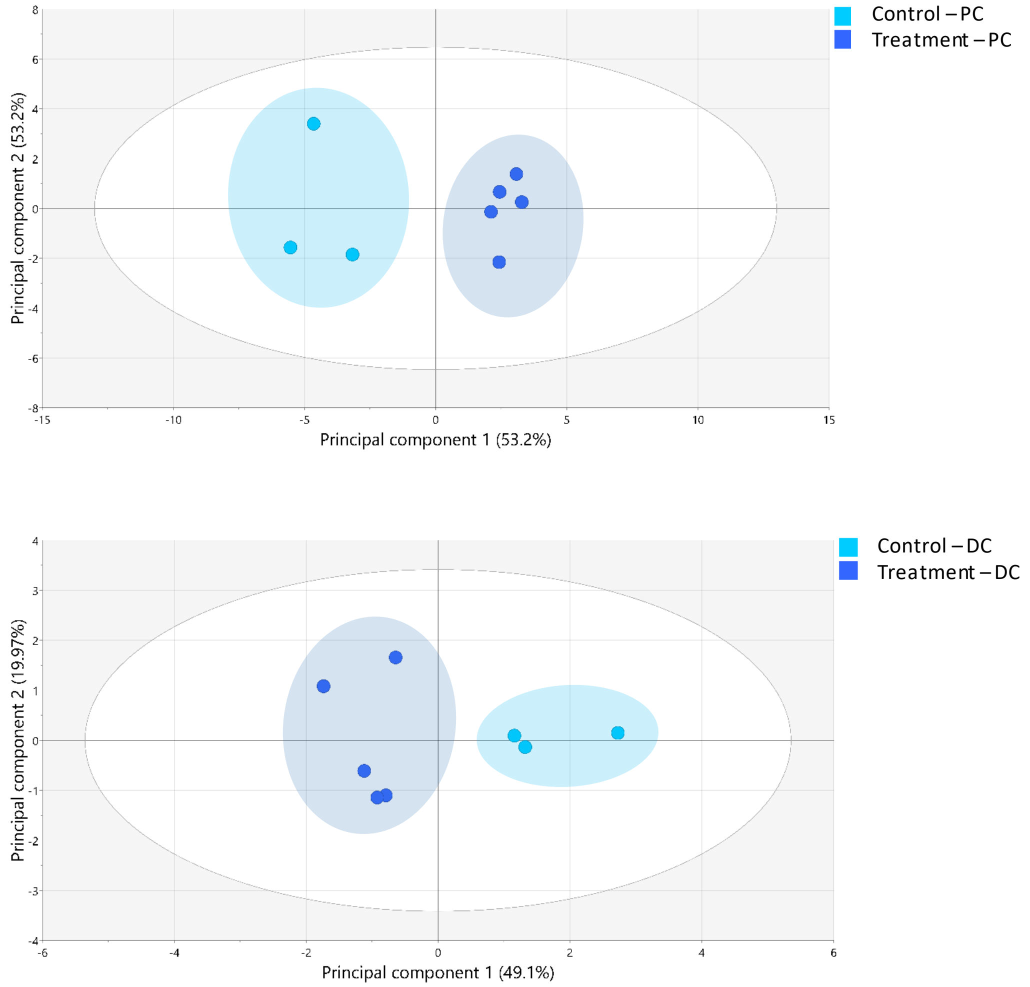

Supplementary Figure 6. The UHPLC-HRMS profiling. The PCA-X score plot as obtained upon UHPLC-HRMS-based **(A)** polar metabolic profiling whereby 184 polar metabolites were identified and quantified; and **(B)** lipidomic profiling whereby 58 lipids were identified and quantified. The associated samples were collected during the control (n = 3 samples during second week = 3) and treatment (n = 2 samples during second week and 3 samples during third week = 5) period in the distal colon (DC) reactors of the SHIME®-experiment following treatment with EpiCor postbiotic. Based on the natural patterning of samples, a differentiation between the treatment (dark blue) and the corresponding control (light blue) was observed.

1.2 Supplementary Tables

Supplementary Table 1. Longitudinal fermentation characteristics. Acetate (mM), propionate (mM), butyrate (mM), ammonium (mg/L) and branched chain fatty acid (bCFA; mM) levels during the two control weeks (C1 and C2) and three treatment weeks (TR1, TR2 and TR3) in the proximal (PC) and distal colon (DC) reactors of the SHIME®-experiment following daily treatment with EpiCor postbiotic. Per week, three samples were collected (A, B and C). Data is presented as single values.

	**Ammonium (mg/L)**		**Acetate (mM)**		**Propionate (mM)**		**Butyrate (mM)**		**bCFA (mM)**	
	**PC**	**DC**	**PC**	**DC**	**PC**	**DC**	**PC**	**DC**	**PC**	**DC**
**C 1 (A)**	226.7	303.5	36.87	49.03	13.30	16.16	8.88	9.03	2.04	2.05
**C 1 (B)**	208.6	274.7	35.75	47.88	13.02	15.74	8.35	9.11	1.98	2.02
**C 1 (C)**	214.8	294.3	32.37	43.40	13.31	16.03	8.77	9.39	2.09	2.16
**C 2 (A)**	218.2	298.5	35.96	46.99	13.73	14.70	9.47	9.26	2.14	2.10
**C 2 (B)**	213.8	315.5	35.16	49.07	13.49	14.97	10.17	9.47	2.15	2.09
**C 2 (C)**	245.8	338.8	32.68	44.90	13.07	12.04	11.70	7.92	2.23	2.25
**TR 1 (A)**	245.7	343.9	32.37	43.66	13.26	12.09	11.83	8.13	2.24	2.25
**TR 1 (B)**	264.1	365.5	35.17	43.27	14.26	12.04	12.41	8.21	2.29	2.26
**TR 1 (C)**	298.6	357.2	33.49	43.82	13.49	12.58	13.17	8.95	2.26	2.32
**TR 2 (A)**	260.3	389.1	33.20	44.99	13.29	13.15	13.90	10.00	2.37	2.41
**TR 2 (B)**	275.7	357.6	33.00	45.13	13.47	13.42	13.40	10.16	2.32	2.42
**TR 2 (C)**	252.3	353.6	33.32	42.78	14.15	13.07	13.42	9.81	2.36	2.43
**TR 3 (A)**	391.4	387.0	36.12	47.10	15.31	14.72	13.24	10.06	2.32	2.49
**TR 3 (B)**	293.0	422.2	35.77	46.30	15.33	14.63	13.39	10.22	2.33	2.48
**TR 3 (C)**	302.4	457.8	34.87	45.91	15.39	14.72	12.81	10.05	2.28	2.44
